# Appendicitis

**Published:** 1899-10

**Authors:** Hugh F. Lorimer

**Affiliations:** Fair Haven, Ohio


### APPENDICITIS.*

BY HUGH F. LORIMER, M.D.,

FAIR HAVEN, OHIO.

The subject announced to-day still claims the attention of the profession very markedly, judging from the number of papers on this particular disease at this meeting. While the committee on program, in arranging the symposium, may have had in mind that the best should come last, I shall be content to flatter myself that a high private in the rear ranks was given first place. If the arranging of the symposium had been left to me, I should have made only one change, and that the paper by Dr. Vance " When Should the Surgeon be Called in a Case of Appendicitis," should follow mine.

I probably will steal some of Dr. Vance's thunder. The last topic is a fitting one to close with, for all positively cured cases of appendicitis end surgically. Some one has said, " a good Indian is a dead Indian "—a safe appendix is one cut off. The physician and surgeon have fought on this disease ever since its discovery, from those highest in authority in medicine to the remotest practician. It is common to see such statements as these: " The pendulum is swinging the other way now as it did in oophorectomy, and sur-

*Read before the Ohio State Medical Society, 1899.

geons are not operating in appendicitis so often as in former years." It is probable surgeons are not operating in *peritonitis* so often as they once did because of such an accumulation of experience in unfavorable results; they think it high time that the physician is learning that unwarranted delay means death, and the surgeon is quite willing to let the responsibility rest where it should. Because a few years ago a lot of *fool* doctors became disciples of Beatty, is there any one to lift his voice against the honest opinion of Wyeth, Morris, Richardson, Murphy, Senn, Ashton *et al.*, because they demand interference when surgical skill is indicated?

It is not right to compare sacrificial surgery to the removal of pathologic conditions menacing life. The subject will continue to be brought before the profession often until every member of it believes that appendicitis exists, and that it is a common disease. When the practician ceases to eye the surgeon as a blood-loving, chance-taking creature with hoofs and horns, but is willing to assist him to decide when the "last resort" opportunity is offered, then one-eighth of the program at a State meeting will not be given to one subject. The general practician should be heard from oftener, for it is likely 99 per cent. of cases first fall into his hands.

Dr. Norman Bridge said that "appendicitis is one of the most frequent and dangerous of inflammatory diseases in or about the peritoneal cavity." This disease is so common that, when excluding zymotic fevers, it is the cause of 80 per cent. of all inflammatory abdominal troubles in the male. This is not meant to show comparison in sexes, but again to emphasize the commonness of the disease. There seems to be a difference of opinion as to frequency in males compared to females. Appendicitis has had a remarkable history as a study, and to America is due the advance made in the development of the study. It was twenty years from the time that Willard Parker began opening perityphlitic abscesses, when, in 1886, Fitz gave us the pathology of typhlitis, perityphlitis and cecitis; and Sands took up the work where Parker left off. McBurney lamented the fact six years ago that Sand's name appeared so seldom with the subject. Those who condemn surgeons for wanting to find new diseases for operation would do well to know something of the honesty of this great man. You will remember that up to about 1890 much was said about typhlitis, perityphlitis and cecitis. Murphy says now that 98 per cent. of inflammatory troubles about the *caput coli* are appendicitis. It is noticeable that almost up to the abandoning of these terms the knowledge of troubles at this location was gained at the post-mortem table; since that time the operating-table furnishes specimens of the disease in all its stages, hence we get recent pathology.

The first thing to learn is that appendicitis is a surgical disease; second, peritonitis is not a disease; third, no such thing exists as idiopathic peritonitis. I believe the time is here when physicians are looking for the cause of peritonitis. It was not uncommon a few years ago to see in the mortality reports of cities peritonitis given very frequently in the list. For four months of the fall and winter of 1897–98 I purposely noted the mortality list in a Cincinnati daily, and there was not one report of peritonitis.

*Pathology.*—" From a bacteriologic aspect the appendix may be regarded as an open test-tube and the retained secretions a culture medium." (Senn.) When an area of infection of any sort in the mucosa or peritoneal covering of the appendix takes place, bacteria at once enter the lymphoid structure and the cellular coats. The stage of exudation begins and the tissues as a result are compressed. If this business went on in the colon there would be plenty of room for swelling and exudation, and there would not be compression. The appendix keeps on swelling, but the point of exit for inflammatory products becomes smaller. Then add to these conditions anemia, because there is only a solitary terminal artery, and we are able to appreciate the aptness of Senn's illustration—a test-tube culture medium; now the infection into the tube (usually mixed), and lastly the tube closed.

*Etiology.*—" The etiology, which was for a long time a subject of contention, is now practically agreed upon. It was very difficult to eradicate from the professional mind the deep-rooted erroneous belief that in a great majority of cases appendicitis was due to foreign bodies admitted into and retained in the appendix, as grape-stones, cherry-stones, fragments of bone, etc. We had four cases of foreign body and 38 per cent. of fecal stone. To indicate the causes of appendicitis, I will use the same classification employed in my article of March 3, 1893:

"1. Simple pus infection, producing the catarrhal variety.

"2. Extensive infection by *bacillus coli communis* or pyogenic microbes, producing gangrene of a greater or less portion of the appendix.

"Pressure atrophy with infection of the appendix: (*a*) by fecal concrement; and (*b*) by foreign bodies.

"4. Retention accumulations: (*a*) from cicatricial contractions, stenosis and obliteration; and (*b*) from occlusion by enterolith or foreign body.

"From the reports of autopsies collected I find that in seventy per cent. of the case there is a perforation of the appendix. Of my own cases in which the appendix was removed there were about eighty perforated.

"Simple catarrhal primary appendicitis, if it exists, is rarely brought to the attention of the physician, and still less frequently to the attention of the surgeon. Of two hundred and seven cases reported, we had but one of this variety. A catarrhal inflammation, where the serious form of appendicitis had previously existed, is not uncommon." (Murphy.)

*Symptoms.* "The symptoms of appendicitis are: (1) a sudden pain in the abdomen; (2) shortly followed by nausea, and perhaps vomiting; (3) local tenderness over the site of the appendix and most frequently in the right iliac region; (4) elevation of temperature. These symptoms occurring in this order, without a previous history of genito-urinary infection, lesions of the gall-tracts or Pott's disease, indicate appendicitis with almost uniform regularity. They do not indicate that the appendix is gangrenous or that it is a simple catarrhal appendicitis. They do not indicate whether the appendix has perforated or has not perforated; nor whether the cause is an infection with the staphylococcus, streptococcus, *bacillus coli communis* or the presence of a fecal stone or a foreign body. They do not indicate whether it is stenosis of the appendix or an *appendicitis obliterans*, but merely that it is an appendicitis, a disease of the appendix." (Murphy.)

To be called at this time to see a patient taken suddenly sick especially with severe pains over the stomach, there would be a routine course as to the subjective examination presenting itself to me. I was impressed some years ago by reading a work by Sir Henry Thompson on urinary diseases with this statement: That there were four questions always to be asked in suspected urinary diseases, and always in regular order, too. And out of these grew all other questions. The same can be said of pain in appendicitis.

1. *Location.* Where is the pain? "Over the stomach," "in the bowels," "through the bowels," "in the bladder," are common answers. A great majority complain of the stomach, and this one thing fools more physicians than any other symptom, and so often leads the attention to at once attempt to confirm the patient's belief that the trouble is in the stomach by the objective sign, palpating the stomach, and next whack out the hypodermic and treat the patient for neuralgia of the stomach—neuralgia of the stomach is an uncommon disease—subjective and objective examination, diagnosis and treatment all in a jiffy.

2. *Character.* Pain is sudden, severe, cutting, sharp, and, as I have heard it described, cruel. The suddenness of the pain is often taken to mean acute indigestion.

3. *Cause.* Inquiry in the majority of cases will tell you they erred in diet or did something out of the ordinary, which they attribute as the cause, and may well say: "I allowed my bowels to become constipated, which always brings on these attacks," an expression in which there is more truth than poetry.

4. *Number of attacks.* If you have not found out by the previous questions as to whether this is a first attack, such wants to be determined before making an examination. This is not always as easy as we might at first suppose. Not with intended evasion on the part of the patient, but for lack of appreciation as to its importance, which cannot be overestimated by the physician. You will be told that the patient had attacks of neuralgia of the stomach, or was threatened with inflammation of the bladder, etc., etc. So much for pain. Vomiting does not last long as a rule; if it does, and there is stercoraceous matter, the case is dangerous. Pulse does not count for much in the very early stages. Temperature is no guide; it is likely the vast majority of cases never reach 101 degrees; fatal cases, proving so in twenty-four to thirty-six hours, may never reach 100 degrees.

Tenderness is elicited by pressure over the base of the appendix; the location is not quite always the same. The tenderness can be found over what is called McBurney's point, which is one and one-half to two inches inner to the superior spinous process of the ilium, on a line drawn from the process to the umbilicus. (It would be better, as individuals differ so in size, to say where the right side of the recti muscles cross the line designated.) This point is not found by shutting the fist and bearing down upon the abdomen anywhere below the umbilicus, but, to use McBurney's own words: "When with one finger you make firm pressure over this point in a case of appendicitis, you will usually find that pain produced is greater at this point than at any other." I would always search for this unawares to the patient. Rigidity of the abdominal muscles, especially of the right side, is a very important sign. Recurring or relapsing appendicitis is applied to cases which occur after first attacks. Recurrent cases are those which come on at intervals of a year or so; relapsing, those attacks which are very close, say every week or two. Symptoms of recurrent attacks are same as those of a primary one. One attack predisposes to another. Hawkins claims twenty-three per cent. and Fitz forty-four per cent. in which there has been a previous attack.

The statement made that appendicitis is purely a surgical disease is not to mean that every case is to be operated upon, but that a surgeon should always be in communication or present in a case that is in any way severe. And note what I say from the medical side of the house.

"We do not know the line between proper medical treatment and the demand for surgical interference, and rather despair of finding it. The medical man is practically powerless to control the destiny of the patient. It is always a surgical disease, and the mortality should be materially lessened by skillful surgical treatment." (Norman Bridge.)

"So impressed am I with the fact that we physicians lose lives by temporizing with many cases of appendicitis, that I prefer in hospital work to have the suspected cases admitted directly to the surgical side. The general practitioner does well to remember whether his leanings be toward the conservative or the radical methods of treatment; that the surgeon is often called too late, never too early. There is no medicinal treatment of appendicitis. There are remedies which will allay the pain, but there are none capable of controlling the course of the disease." (Osler.)

"As soon as the diagnosis is established—indeed, pending its settlement—a competent surgeon should be associated with the physician for the reason that in the vast majority of cases operative treatment is sooner or later demanded, while the hour for such treatment is best settled by daily conference. The course of cases of appendicitis is often delusive, and the surgeon who operates is frequently more likely to have seen more cases than the physician. The diagnosis being established, operative treatment should be recommended, except in cases where the disease is so far advanced as to make it unlikely that the patient will be saved by operation. My reason for this belief is that while a majority of cases of simple appendicitis may subside with rest, in a very large number, at least 25 per cent., the primary attack leaves the patient predisposed to another, at once more severe and dangerous than the first, while we have no guarantee that any attack will subside without suppuration, or what is worse, without leaving the condition referred to in which malignant inflammation or perforation may set in at any moment without warning." (Tyson.)

The consensus of opinion on the surgical side is, that when a diagnosis is made in the acute form, if there is not much improvement in the pain, pulse no better, temperature continues above normal, and tenderness is marked, operate in thirty-six or forty-eight hours. Osler puts it at the outside seventy-two hours. The medical treatment must be meted out according to each case, and in each case right early we conclude that "a condition and not a theory confronts us."

Rest in bed first.

A great majority of cases get over the attack with free purgation and enemas, while in some anything that brings about peristalsis of the bowels hastens the rupture of an abscess into the peritoneal cavity, followed with peritonitis, and yet if peritonitis should announce itself salines are to be administered with a flush hand.

The physician and surgeon have crossed swords in the use of opium in these cases, the physician often administering opium not only until the patient is easier, but rendering him quite comfortable; narcotizing his own judgment in the case, benumbing the fears of the friends, both physician and friends to be aroused in a few days to realize that the case has proved fatal. The surgeon, on the other hand, is not content with letting the spirit of Alonzo Clark rest, but attempts to drive one more nail in his coffin by giving no opium. It seems to me there are some cases in which the pain is so extreme that the hypodermic injection of morphine is not only indicated as a means of relief, but for its diagnostic value. Though with a sparing hand opium should be given, I should despair of repeating the dose without advisement of surgeon. Hot fomentations are to be used; if tumefaction is present I believe the ice-bag better until surgical aid comes to our hand. I have seen twenty-six cases of appendicitis in my practice and in consultation the last seven years, mention of which will be made in discussion of other papers.

CONCLUSIONS BY ASHTON.

" 1. That as we have no exact means of determining the pathologic changes going on we must consider all cases of appendicitis dangerous from the beginning.

" 2. That to wait for evidence of perforation of appendix, pus, bowel obstruction or peritonitis is unwarranted delay.

" 3. That marked local tenderness over McBurney's point, continued elevation of temperature, rapid pulse, and tumefaction are conditions indicating immediate operation.

" 4. That in mild cases, with a positive diagnosis, operation is demanded in forty-eight hours if no improvement has taken place.

" 5. That all cases should be operated upon as soon as the diagnosis is clear."

